# Pathologic response and safety of preoperative treatment regimens in gastric cancer undergoing D2 gastrectomy: a real-world cohort study

**DOI:** 10.3389/fimmu.2026.1766793

**Published:** 2026-01-29

**Authors:** Penghui Liu, Na Li, Jiwu Guo, Jizhen Wang, Zhen Wang, Gengyutong Zhao, Ziyuan Mou, Jie Mao

**Affiliations:** 1 Lanzhou University Second Clinical Medical College, Lanzhou, China; 2 Lanzhou University Second Hospital, Lanzhou, China; 3 Northeastern University, Boston, MA, United States

**Keywords:** conversion therapy, gastric cancer, immunotherapy, neoadjuvant therapy, pathological response, PD-L1

## Abstract

**Objective:**

To compare pathological response and safety among three preoperative treatment regimens—chemotherapy alone, chemotherapy plus immunotherapy, and chemotherapy plus immunotherapy and targeted therapy—in gastric cancer undergoing D2 gastrectomy, and to assess biomarker associations with response.

**Methods:**

We retrospectively included 195 patients who received preoperative systemic therapy followed by D2 gastrectomy: chemotherapy alone (n = 47), chemotherapy plus immunotherapy (n = 100), or chemotherapy plus immunotherapy and targeted therapy (n = 48). Preoperative therapy was considered neoadjuvant for cM0 disease and conversion therapy for selected baseline cM1 disease. The primary endpoint was MPR (including pCR). pCR was also reported separately as a subset of MPR; secondary endpoints were preoperative treatment-related adverse events, surgical and pathological outcomes, and disease-free and overall survival (DFS, OS). Logistic regression was used to identify factors associated with MPR; DFS and OS were evaluated with Kaplan–Meier and Cox models.

**Results:**

Overall pCR and MPR rates were 20.5% and 40.0%. MPR rates were 27.7%, 37.0%, and 58.3% in the chemotherapy, chemotherapy+immunotherapy, and triple-regimen groups. The triple regimen improved MPR versus chemotherapy in unadjusted analysis, but regimen type was not an independent predictor after adjustment. Lauren intestinal subtype and PD-L1 CPS ≥5 were strongly associated with higher MPR, and in PD-L1–low tumors the triple regimen was associated with higher MPR. Rates of grade ≥3 adverse events and postoperative complications were similar across groups. DFS and OS did not differ significantly; higher cN stage and older age were associated with worse outcomes.

**Conclusion:**

Preoperative treatment regimens containing immunotherapy, particularly the triple combination, improved pathological response without increasing preoperative risk. Tumor biology—especially Lauren subtype and PD-L1 expression—had a greater impact on response than regimen intensity, supporting biomarker-guided strategies.

## 1 Introduction

Gastric cancer remains one of the leading causes of cancer-related mortality worldwide, with a particularly high burden in East Asia (1, 2). For patients with advanced disease, gastrectomy alone is usually insufficient, and preoperative chemotherapy has become a standard component of multidisciplinary treatment, improving R0 resection rates and survival compared with surgery alone (3–5). However, even with standard preoperative chemotherapy, long-term outcomes remain unsatisfactory and early postoperative recurrence is common, underscoring the need to further optimize preoperative systemic therapy.

Immune checkpoint inhibitors have reshaped first-line systemic therapy for advanced or metastatic gastric cancer, and adding programmed death 1 (PD-1) inhibitors to platinum-based chemotherapy has been incorporated into treatment guidelines (5, 6). On this basis, multiple phase II/III trials are evaluating chemotherapy plus immunotherapy, with or without targeted agents, in the preoperative systemic setting and have reported improved pathological response compared with chemotherapy alone (7–9). However, the optimal regimen, patient selection, and real-world safety of intensified approaches remain uncertain. In parallel, targeted agents are increasingly combined with chemotherapy and/or immunotherapy in biologically selected patients, leading to a spectrum of preoperative treatment regimens in clinical practice—from chemotherapy alone (Chemo), to chemotherapy plus immunotherapy (Chemo+IO), to chemotherapy plus immunotherapy and targeted therapy (Chemo+IO+Targeted) (10, 11). Yet, in relatively homogeneous surgical cohorts undergoing D2 gastrectomy, direct real-world comparisons of these different preoperative treatment strategies are still limited.

Biomarkers including human epidermal growth factor receptor 2 (HER2), microsatellite instability (MSI), and programmed death-ligand 1 (PD-L1) have been widely used to guide systemic therapy in advanced gastric cancer, but their predictive and prognostic value in the preoperative therapy era of diverse combination regimens is not fully defined (5, 11, 12). Real-world data integrating detailed clinicopathologic features, preoperative treatment regimens, and biomarker profiles may help identify patients who derive additional benefit from intensified therapy beyond conventional chemotherapy.

Against this background, we conducted a real-world retrospective cohort study of patients with gastric cancer treated with three preoperative treatment strategies followed by D2 gastrectomy. The primary objective was to compare pathological response among the three regimens. Secondary objectives were to evaluate preoperative treatment exposure and safety, surgical and postoperative pathological outcomes, and early disease-free survival (DFS) and overall survival (OS). Additionally, exploratory analyses of HER2, MSI, and PD-L1 were performed to examine their association with pathological response and to explore the potential modifying effect of PD-L1 on the efficacy of different preoperative treatment strategies.

## 2 Methods

### 2.1 Study design and patient selection

This was a single-center, retrospective, real-world cohort study. We collected clinical data of patients with gastric cancer who received preoperative systemic therapy and subsequently underwent gastrectomy with D2 lymphadenectomy.

For patients with non-metastatic disease (cM0) at baseline, preoperative systemic therapy was defined as neoadjuvant therapy. For selected patients with baseline distant metastasis (cM1) who were considered potentially convertible and proceeded to surgery only after radiologic response and multidisciplinary reassessment confirmed feasibility of curative-intent resection, preoperative systemic therapy was defined as conversion therapy. Baseline cM1 disease was considered potentially resectable/convertible only when metastatic burden was limited (oligometastatic) and judged amenable to curative-intent resection after systemic therapy by the MDT. Patients proceeded to D2 gastrectomy only if restaging showed no progression (no new lesions) and the MDT confirmed feasibility of R0 resection.

Inclusion Criteria: (1) Pathologically confirmed gastric cancer. (2) Radiologic and endoscopic evidence of potentially resectable/convertible disease, with MDT confirmation of suitability for curative-intent surgery after preoperative systemic therapy (neoadjuvant or conversion intent as defined above). (3) Receipt of preoperative systemic therapy at our institution. (4) Underwent gastrectomy with D2 lymphadenectomy. (5) Availability of complete baseline clinicopathologic data and treatment records. Exclusion Criteria: (1) No preoperative systemic therapy. (2) No surgical resection. (3) Presence of other malignancies potentially affecting prognosis. (4) Missing key data for eligibility or primary endpoint (e.g., baseline staging, regimen information, pathological response). A total of 195 patients were ultimately included.

### 2.2 Preoperative systemic therapy

Regimen decision-making and clinical workflow. This was a single-center, retrospective real-world cohort; therefore, no prespecified institutional protocol mandated a single uniform preoperative regimen. Treatment selection was individualized by the attending physicians (with multidisciplinary team [MDT] discussion when appropriate) and followed a structured workflow in routine practice. After identification of a gastric space-occupying lesion, endoscopic biopsy was performed for histopathologic confirmation, and pretreatment biomarker testing (HER2, PD-L1, and MSI) on biopsy specimens was obtained when feasible. Regimen decisions integrated: (1) baseline disease status and treatment intent (neoadjuvant for cM0; conversion intent for selected baseline cM1 patients as defined above); (2) tumor burden and clinical stage; (3) biomarker results when available; (4) patient fitness (age, ECOG performance status, comorbidities, and organ function); (5) contraindications or intolerance to immune checkpoint inhibitors and/or targeted agents; and (6) shared decision-making incorporating patient preference, drug accessibility/reimbursement, and financial considerations. In routine practice, biomarker results may be unavailable in a small proportion of cases or not available at the time of initial regimen initiation; therefore, treatment escalation (e.g., adding immunotherapy or a targeted agent) could also be informed by interval response assessment, toxicity tolerance, and updated MDT reassessment.

Chemotherapy backbone (all groups). Chemotherapy served as the backbone for all regimen groups and consisted primarily of platinum plus fluoropyrimidine combinations, mainly SOX (S-1 plus oxaliplatin) and FLOT (docetaxel plus 5-fluorouracil/leucovorin/oxaliplatin). Selection between SOX and FLOT was made pragmatically based on patient fitness/tolerance, anticipated perioperative risk, and real-world accessibility.

Chemo group (chemotherapy alone). Chemotherapy alone was generally selected when immunotherapy and/or targeted therapy was not appropriate, was not feasible, was contraindicated, or was declined by the patient after shared decision-making.

Chemo+IO group (chemotherapy plus immunotherapy). Chemo+IO was considered for patients without major contraindications to immunotherapy (e.g., conditions conferring unacceptable risk of immune-related toxicity as judged clinically; active autoimmune disease requiring systemic immunosuppression; prior severe irAEs). Immune checkpoint inhibitors were administered intravenously every 2–3 weeks according to the agent label and contemporary clinical practice. Given the Chinese real-world context, multiple PD-1 inhibitors (and a PD-1/CTLA-4 bispecific antibody) were used depending on availability, reimbursement/access, and physician experience; the specific ICI agents and their distributions are summarized in **Supplementary Table S2**.

Chemo+IO+Targeted group (chemotherapy plus immunotherapy plus targeted therapy). Chemo+IO+Targeted was used in biologically or clinically selected patients in whom adding a targeted agent was deemed appropriate based on biomarker status and/or clinical risk assessment. Targeted therapy comprised two clinically relevant categories: (1) anti-HER2 therapy, preferentially selected for tumors with HER2 positivity on pretreatment biopsy (as defined in Section 2.5), and (2) anti-angiogenic therapy, considered in selected patients based on clinical factors (e.g., tumor burden/advanced stage and risk–benefit evaluation), while accounting for safety-relevant considerations (e.g., bleeding risk, thrombotic history, hypertension control, and perioperative risk). Because targeted agents were heterogeneous and patient numbers for each specific agent were limited, the Chemo+IO+Targeted group was analyzed as a single category; thus, no agent-specific efficacy conclusions are intended. To improve transparency and address indication concerns, the exact targeted agents used and their distributions (including anti-HER2 and anti-angiogenic subtypes) are provided in **Supplementary Table S2**.

Treatment exposure and safety capture. We recorded the number of preoperative cycles, treatment duration (from treatment initiation to surgery), adverse events (AEs; including any-grade AEs, grade ≥3 AEs, immune-related AEs, and serious AEs), and any dose reductions, interruptions, or permanent discontinuations attributable to AEs. Postoperative treatments, when applicable, were recorded but were not used to define the preoperative regimen groups.

### 2.3 Surgical Procedure and postoperative Care

After completion of preoperative treatment, all patients underwent curative gastrectomy, including total, distal, or proximal. All procedures followed contemporary guidelines and institutional protocols, with standard D2 lymphadenectomy performed in all cases.

Collected type of gastrectomy, margin status (R0/R1), length of hospital stay, and postoperative complications (including abdominal distension, bleeding, intra-abdominal or pleural effusion, anastomotic leak, wound infection, and pulmonary infection).

### 2.4 Pathological evaluation

Recorded variables included Lauren classification (intestinal, diffuse, mixed), Histologic grade (Moderate, Moderate-Low, Low), Borrmann type, and postoperative pathological stage (ypT, ypN) based on the AJCC 8th edition. The total number of examined lymph nodes and the number of positive nodes were documented, and preoperative clinical staging (cT/N) was compared with ypT/N to assess T and N downstaging or progression.

Tumor regression was graded using the Becker Tumor Regression Grade (TRG): TRG 1 (1a/1b) as marked regression, TRG 2 as partial regression, and TRG 3 as poor regression. Pathological complete response (pCR) was defined as no residual viable tumor in both the primary site and all resected lymph nodes (TRG 1a). Major pathological response (MPR) was defined as ≤10% residual viable tumor in the primary tumor (TRG 1a/1b).

### 2.5 Biomarker Testing

HER2, MSI, and PD-L1 were assessed on pre-treatment tumor biopsy specimens. HER2 positivity was defined as IHC 3+ or IHC 2+ with gene amplification; all other results were classified as HER2-negative. MSI status was categorized as MSI-high (MSI-H) or microsatellite stable (MSS). PD-L1 expression was reported as combined positive score (CPS) and analyzed dichotomously as CPS <5 vs CPS ≥5.

### 2.6 Endpoint definitions

The primary endpoint was MPR (including pCR). pCR (TRG 1a) was also reported separately as a subset of MPR. For the main analyses, pathological response was dichotomized as MPR vs non-MPR.

Secondary endpoints included: (1) preoperative treatment exposure and safety: number of treatment cycles, time from initiation of treatment to surgery, any AEs, grade ≥3 AEs, irAEs, SAEs, and AE-related dose reduction, interruption, or permanent discontinuation. (2) Surgical and short-term outcomes: type of gastrectomy, R0 resection rate, postoperative complications, and length of hospital stay. (3) Postoperative pathological characteristics: distribution of Becker TRG, ypT/ypN stage, lymph node retrieval and metastasis, and T/N downstaging. (4) Follow-up outcomes: disease-free survival (DFS) was defined as the interval from gastrectomy to first local recurrence, distant metastasis, disease progression, or death from any cause, whichever occurred first; patients without an event were censored at last follow-up. overall survival (OS) was defined as the interval from gastrectomy to death from any cause, with survivors censored at the date of last contact.

### 2.7 Statistical analysis

All statistical analyses were performed using SPSS (version 26.0, IBM Corp., Armonk, NY, USA). Continuous variables were presented as medians with interquartile ranges (IQR), and compared using the Kruskal–Wallis test. Categorical variables were presented as frequencies, and compared using the χ² test.

For the primary endpoint (MPR vs non-MPR), univariate logistic regression was first performed with the Chemo as the reference. A multivariable logistic regression model was then constructed including treatment regimen, sex, age, ECOG status, tumor location, Lauren classification, and clinical T, N, and M stage. Results are reported as odds ratios (ORs) with 95% confidence intervals (CIs) and illustrated using forest plots. A sensitivity analysis was performed excluding patients with baseline distant metastasis (cM1) who were treated with conversion intent.

An extended model additionally incorporated HER2 status, MSI status, and PD-L1 expression to evaluate the influence of these biomarkers. Multicollinearity was assessed using variance inflation factors (VIF), and no relevant multicollinearity was detected.

Exploratory subgroup analyses were conducted stratified by PD-L1 CPS (<5 vs ≥5). Within each stratum, logistic regression was used to estimate ORs and 95% CIs for treatment regimen. Additionally, a model including treatment regimen, PD-L1 status, and a regimen × PD-L1 interaction term was fitted to test effect modification by PD-L1. These biomarker-related and subgroup analyses were considered exploratory and hypothesis-generating, and were not adjusted for multiple testing. Given the modest sample size and small numbers within PD-L1 strata, the regimen × PD-L1 interaction test was underpowered; therefore, interaction findings should be interpreted as hypothesis-generating.

For survival outcomes, Kaplan–Meier curves for DFS and OS were plotted for the three groups and compared using the log-rank test. When estimable, median DFS/OS with 95% CIs was reported. Multivariable Cox proportional hazards models were constructed for DFS and OS. Results were presented as hazard ratios (HRs) with 95% CIs. For graphical display only, survival curves were truncated at 36 months; all statistical tests and Cox models were based on the full follow-up data.

All analyses were performed using available data, with listwise deletion for missing values in the corresponding models; no imputation was applied. No formal correction for multiple comparisons was undertaken. Aside from the prespecified primary endpoint analysis, all other findings—particularly biomarker-related and subgroup results—should be interpreted with caution. A two-sided p-value <0.05 was considered statistically significant.

## 3 Results

### 3.1 Baseline characteristics of patients

A total of 195 patients were included in the study: 47 patients in the Chemo group, 100 in the Chemo+IO group, and 48 in the Chemo+IO+Targeted group. The median age of the patients was approximately 59 years, the median BMI was 22.9 kg/m², with the majority being male (86.7%) and having an ECOG status of 0–1 (92.3%). There were no significant differences between the groups for these baseline characteristics.

The tumors were primarily located in the cardia (35.9%), antrum (34.4%), and gastric body (29.7%). Borrmann type III accounted for 84.6%, and type IV for 9.2%. cT3–4 stage was observed in 96.4%, cN2–3 in 69.2%, and cM1 in 8.7%. These baseline cM1 patients underwent preoperative systemic therapy with conversion intent and proceeded to D2 gastrectomy after multidisciplinary reassessment. HER2 positivity was found in 12.8%, MSI-H in 3.6%, and PD-L1 (CPS ≥5) in 42.1%. No significant differences were observed among the three groups. The only statistically significant difference was in Lauren classification (p = 0.021): 49.2% of patients had intestinal type, 36.4% had diffuse type, and 14.4% had mixed type. The Chemo+IO+Targeted group had a relatively higher proportion of intestinal type, while the chemotherapy group had a higher proportion of diffuse type (**Table 1**).

**Table 1:** Baseline clinicopathologic characteristics by preoperative treatment regimen.

Variable		Total (n=195)	Chemo (n=47)	Chemo+IO (n=100)	Chemo+IO+Targeted (n=48)	*p*
Age, year, median (IQR)		59.0 (54.0,66.0)	59.0 (56.0,64.5)	60.0 (53.8,67.2)	57.5 (53.8,62.8)	0.457
BMI, kg/m², median (IQR)		22.9 (22.2,23.7)	22.8 (21.9,23.7)	22.9 (22.4,23.6)	23.1 (22.5,24.0)	0.137
Sex, n (%)	Male	169 (86.7%)	42 (89.4%)	88 (88.0%)	39 (81.2%)	0.434
	Female	26 (13.3%)	5 (10.6%)	12 (12.0%)	9 (18.8%)	
ECOG status, n (%)	0	17 (8.7%)	4 (8.5%)	10 (10.0%)	3 (6.2%)	0.934
	1	163 (83.6%)	39 (83.0%)	82 (82.0%)	42 (87.5%)	
	2	15 (7.7%)	4 (8.5%)	8 (8.0%)	3 (6.2%)	
Tumor location, n (%)	cardia	70 (35.9%)	14 (29.8%)	43 (43.0%)	13 (27.1%)	0.302
	body	58 (29.7%)	16 (34.0%)	27 (27.0%)	15 (31.2%)	
	antrum	67 (34.4%)	17 (36.2%)	30 (30.0%)	20 (41.7%)	
Lauren classification, n (%)	intestinal	96 (49.2%)	15 (31.9%)	49 (49.0%)	32 (66.7%)	0.021
	diffuse	71 (36.4%)	23 (48.9%)	37 (37.0%)	11 (22.9%)	
	mixed	28 (14.4%)	9 (19.1%)	14 (14.0%)	5 (10.4%)	
Histologic grade, n (%)	Moderate Grading	5 (2.6%)	0 (0.0%)	2 (2.0%)	3 (6.2%)	0.227
	Moderate-Low Grading	111 (56.9%)	27 (57.4%)	54 (54.0%)	30 (62.5%)	
	Low Grading	79 (40.5%)	20 (42.6%)	44 (44.0%)	15 (31.2%)	
Borrmann type, n (%)	II	12 (6.2%)	1 (2.1%)	7 (7.0%)	4 (8.3%)	0.585
	III	165 (84.6%)	40 (85.1%)	84 (84.0%)	41 (85.4%)	
	IV	18 (9.2%)	6 (12.8%)	9 (9.0%)	3 (6.2%)	
Clinical T stage, n (%)	cT2	7 (3.6%)	1 (2.1%)	5 (5.0%)	1 (2.1%)	0.249
	cT3	94 (48.2%)	17 (36.2%)	52 (52.0%)	25 (52.1%)	
	cT4	94 (48.2%)	29 (61.7%)	43 (43.0%)	22 (45.8%)	
Clinical N stage, n (%)	cN0	15 (7.7%)	6 (12.8%)	4 (4.0%)	5 (10.4%)	0.263
	cN1	45 (23.1%)	9 (19.1%)	28 (28.0%)	8 (16.7%)	
	cN2	89 (45.6%)	24 (51.1%)	43 (43.0%)	22 (45.8%)	
	cN3	46 (23.6%)	8 (17.0%)	25 (25.0%)	13 (27.1%)	
Clinical M stage, n (%)	cM0	178 (91.3%)	42 (89.4%)	92 (92.0%)	44 (91.7%)	0.864
	cM1	17 (8.7%)	5 (10.6%)	8 (8.0%)	4 (8.3%)	
HER2 status, n (%)	Negative	170 (87.2%)	43 (91.5%)	88 (88.0%)	39 (81.2%)	0.309
	Positive	25 (12.8%)	4 (8.5%)	12 (12.0%)	9 (18.8%)	
MSI status, n (%)	MSS	188 (96.4%)	47 (100.0%)	96 (96.0%)	45 (93.8%)	0.249
	MSI-H	7 (3.6%)	0 (0.0%)	4 (4.0%)	3 (6.2%)	
PD-L1 status, n (%)	CPS <5	113 (57.9%)	33 (70.2%)	54 (54.0%)	26 (54.2%)	0.148
	CPS ≥5	82 (42.1%)	14 (29.8%)	46 (46.0%)	22 (45.8%)	

Data are presented as median (IQR) or n (%). p values refer to overall comparisons among the three groups (Kruskal–Wallis test for continuous variables; χ² test for categorical variables).

### 3.2 Treatment Process and Adverse Events

The median number of preoperative treatment cycles in the whole cohort was 4 (IQR 3–4). The median interval from initiation of therapy to surgery was approximately 14 weeks, with a slightly longer interval in the Chemo group; no significant differences were observed across groups.

The overall incidence of any-grade AEs was 96.4%, and was comparable across groups (95.0%–97.9%, p = 0.554). Grade ≥3 AEs occurred in 37.4% of patients (29.8%, 38.0%, and 43.8%, respectively; p = 0.367). The overall incidence of SAEs was 12.3%, without differences across groups. The proportions requiring dose reduction or interruption (30.3%) and permanent discontinuation (5.6%) due to AEs were comparable across groups.

IrAEs occurred almost exclusively in patients receiving immunotherapy: no irAEs were observed in the Chemo group, whereas 20.0% in the Chemo+IO group and 25.0% in the Chemo+IO+Targeted group, with a significant difference among the three groups (p = 0.002; **Supplementary Table S1**).

### 3.3 Surgical procedures and postoperative outcomes

All patients successfully underwent curative gastrectomy. Overall, 52.3% received total gastrectomy, 34.4% distal gastrectomy, and 13.3% proximal gastrectomy (p = 0.094). The overall R0 resection rate was 99.5%, with only one R1 resection, and was comparable across groups. The median postoperative hospital stay was 14 days (IQR 12.0–16.5); patients in the Chemo group had a slightly longer stay than those in the Chemo+IO and Chemo+IO+Targeted groups, although the difference was not statistically significant (p = 0.082).

Postoperative complications occurred in 38.5% of patients (75/195): 36.2% (17/47) in the Chemo group, 39.0% (39/100) in the Chemo+IO group, and 39.6% (19/48) in the Chemo+IO+Targeted group (p = 0.930). The most common complications were abdominal distension (16.4%), pulmonary infection (11.3%), and intra-abdominal effusion (10.3%). Pleural effusion, wound infection, anastomotic leakage, and postoperative bleeding were less frequent (each 3%–7%), with similar distributions across groups (p > 0.05). Preoperative treatment regimens did not significantly influence postoperative complication rates or surgical risk (**Table 2**).

**Table 2:** Surgical procedures, hospital stay, and postoperative complications by regimen.

Variable		Total (n=195)	Chemo (n=47)	Chemo+IO (n=100)	Chemo+IO+Targeted (n=48)	*p*
Surgery and hospital Status	Type of gastrectomy, n (%)					0.094
	total	102 (52.3%)	27 (57.4%)	48 (48.0%)	27 (56.2%)	
	distal	67 (34.4%)	17 (36.2%)	32 (32.0%)	18 (37.5%)	
	proximal	26 (13.3%)	3 (6.4%)	20 (20.0%)	3 (6.2%)	
	Resection margin status, n (%)					0.620
	R0	194 (99.5%)	47 (100.0%)	99 (99.0%)	48 (100.0%)	
	R1	1 (0.5%)	0 (0.0%)	1 (1.0%)	0 (0.0%)	
	Length of hospital stay, days, median (IQR)	14.0 (12.0,16.5)	15.0 (13.0,17.0)	13.5 (11.0,16.0)	14.0 (11.8,16.0)	0.082
Complications	Any complication, n (%)	75 (38.5%)	17 (36.2%)	39 (39.0%)	19 (39.6%)	0.930
	Abdominal distension, n (%)	32 (16.4%)	7 (14.9%)	17 (17.0%)	8 (16.7%)	0.948
	Bleeding, n (%)	10 (5.1%)	2 (4.3%)	5 (5.0%)	3 (6.2%)	0.904
	Peritoneal effusion, n (%)	20 (10.3%)	3 (6.4%)	12 (12.0%)	5 (10.4%)	0.578
	Pleural effusion, n (%)	10 (5.1%)	0 (0.0%)	7 (7.0%)	3 (6.2%)	0.184
	Anastomotic fistula, n (%)	8 (4.1%)	2 (4.3%)	4 (4.0%)	2 (4.2%)	0.997
	Wound infection, n (%)	14 (7.2%)	3 (6.4%)	7 (7.0%)	4 (8.3%)	0.930
	Pulmonary infection, n (%)	22 (11.3%)	5 (10.6%)	12 (12.0%)	5 (10.4%)	0.948
	Other complications, n (%)	5 (2.6%)	0 (0.0%)	3 (3.0%)	2 (4.2%)	0.405

Data are presented as median (IQR) or n (%). p values refer to overall comparisons among the three groups (Kruskal–Wallis test for continuous variables and χ² test for categorical variables).

### 3.4 Postoperative pathological staging and pathological response

Overall, 50.8% of patients had ypT0–2 and 57.4% had ypN0. The distributions of ypT and ypN did not differ significantly among the three groups (p = 0.552 and p = 0.460), suggesting comparable degrees of tumor and nodal downstaging (**Table 3**). Clinical–pathological downstaging was achieved in 80.5% of patients, whereas 13.3% (26/195) showed radiologic or pathological progression, with similar proportions across regimens.

**Table 3:** Multivariable logistic regression for factors associated with pathologic response.

Variable	OR	95% CI	p
Regimen: chemo+IO vs chemo	1.09	(0.41–2.90)	0.870
Regimen: chemo+IO+targeted vs chemo	2.00	(0.66–6.06)	0.221
Sex: Male vs Female	1.05	(0.35–3.21)	0.928
Location: Antrum vs Body	1.01	(0.39–2.59)	0.986
Location: Cardia vs Body	0.68	(0.26–1.77)	0.426
Lauren: Diffuse vs Intestinal	0.05	(0.02–0.13)	<0.001
Lauren: Mixed vs Intestinal	0.05	(0.01–0.18)	<0.001
Age	1.01	(0.96–1.05)	0.809
ECOG status	0.43	(0.17–1.07)	0.070
cT	0.97	(0.48–1.95)	0.929
cN	1.02	(0.65–1.60)	0.928
cM:M1 vs M0	2.14	(0.56–8.20)	0.265

Outcome variable was pathologic response defined as MPR (MPR vs. no-MPR). ORs are adjusted for all variables listed in the table, with the Chemo group as the reference.

According to the Becker grading system, TRG 1 (complete/near-complete regression) was observed in 40.0% of patients, TRG 2 in 26.7%, and TRG 3 in 33.3%. The Chemo+IO+Targeted group had the highest proportion of TRG 1 (58.3%), followed by 37.0% in the Chemo+IO group and 27.7% in the Chemo group (p = 0.026). Correspondingly, the overall pCR, and MPR rates were 20.5%, and 40.0%, respectively. The MPR rates increased stepwise across the three regimens—27.7%, 37.0%, and 58.3% in the Chemo, Chemo+IO, and Chemo+IO+Targeted groups (p = 0.006). The median number of retrieved lymph nodes was 23 (IQR 17–28) for the whole cohort, with a slightly higher median in the Chemo+IO+Targeted group compared with the Chemo and Chemo+IO groups (24 vs 21 and 24, p = 0.041). The median number of positive lymph nodes was 0 (IQR 0–2) in all groups, with no significant between-group differences (**Table 4**).

**Table 4:** Pathologic outcomes and downstaging by preoperative treatment regimen.

Variable	Total (n=195)	Chemo (n=47)	Chemo+IO (n=100)	Chemo+IO+Targeted (n=48)	*p*
ypT, n (%)					0.552
ypT0	41 (21.0%)	6 (12.8%)	21 (21.0%)	14 (29.2%)	
ypT1	30 (15.4%)	6 (12.8%)	15 (15.0%)	9 (18.8%)	
ypT2	28 (14.4%)	7 (14.9%)	17 (17.0%)	4 (8.3%)	
ypT3	64 (32.8%)	18 (38.3%)	32 (32.0%)	14 (29.2%)	
ypT4	32 (16.4%)	10 (21.3%)	15 (15.0%)	7 (14.6%)	
ypN, n (%)					0.460
ypN0	112 (57.4%)	25 (53.2%)	54 (54.0%)	33 (68.8%)	
ypN1	39 (20.0%)	13 (27.7%)	21 (21.0%)	5 (10.4%)	
ypN2	19 (9.7%)	4 (8.5%)	11 (11.0%)	4 (8.3%)	
ypN3	25 (12.8%)	5 (10.6%)	14 (14.0%)	6 (12.5%)	
TRG_Becker, n (%)					0.026
TRG 1	78 (40.0%)	13 (27.7%)	37 (37.0%)	28 (58.3%)	
TRG 2	52 (26.7%)	17 (36.2%)	28 (28.0%)	7 (14.6%)	
TRG 3	65 (33.3%)	17 (36.2%)	35 (35.0%)	13 (27.1%)	
Pathological Response, n (%)					
pCR	40 (20.5%)	6 (12.8%)	20 (20.0%)	14 (29.2%)	0.036
No	117 (60.0%)	34 (72.3%)	63 (63.0%)	20 (41.7%)	
MPR	78 (40.0%)	13 (27.7%)	37 (37.0%)	28 (58.3%)	0.006
Total LN Count, Median (IQR)	23.0 (17.0,28.0)	21.0 (17.0,24.0)	24.0 (18.0,29.0)	24.0 (17.0,30.0)	0.041
Positive LN Count, Median (IQR)	0.0 (0.0,2.0)	0.0 (0.0,2.0)	0.0 (0.0,2.2)	0.0 (0.0,2.0)	0.409
Downgrading, n (%)					0.972
Yes	157 (80.5%)	38 (80.9%)	80 (80.0%)	39 (81.2%)	
No	12 (6.2%)	2 (4.3%)	7 (7.0%)	3 (6.2%)	
Progression	26 (13.3%)	7 (14.9%)	13 (13.0%)	6 (12.5%)	

Data are presented as n (%) or median (IQR). p values refer to overall comparisons among the three groups (Kruskal–Wallis test for continuous variables and χ² test for categorical variables).

### 3.5 Factors associated with pathological response

In univariate logistic regression using the Chemo group as reference, the OR for achieving MPR was 1.54 (95% CI 0.72–3.28, p = 0.267) in the Chemo+IO group and 3.66 (95% CI 1.55–8.64, p = 0.003) in the Chemo+IO+Targeted group, indicating a significantly higher pathological response rate with the triple regimen before adjustment (**Supplementary Table S2**).

In the multivariable model adjusted for age, sex, tumor location, Lauren classification, ECOG status, and cTNM stage (**Table 3**, **Supplementary Figure S1**), the associations for regimen type were attenuated and no longer significant: OR 1.09 (95% CI 0.41–2.90, p = 0.870) for Chemo+IO and 2.00 (95% CI 0.66–6.06, p = 0.221) for Chemo+IO+Targeted vs Chemo.

In contrast, Lauren classification emerged as the strongest independent predictor of pathological response. Compared with intestinal-type tumors, diffuse-type and mixed-type tumors had markedly lower odds of achieving MPR, with ORs of 0.05 (95% CI 0.02–0.13) and 0.05 (95% CI 0.01–0.18), respectively (both p < 0.001), indicating substantially poorer responses to preoperative treatment. ECOG status showed a non-significant trend toward lower response with worsening performance (OR per 1-point increase 0.43, 95% CI 0.17–1.07, p = 0.070). Age, cT stage, cN stage, cM stage, and tumor location were not significantly associated with MPR (**Table 3**). After excluding baseline cM1 patients (n=17), the between-group difference in MPR remained significant, and the multivariable results were materially unchanged (**Supplementary Table S1**).

### 3.6 Extended model with inclusion of biomarkers

In the extended multivariable logistic regression model incorporating HER2, MSI, and PD-L1, the associations between regimen type and MPR were further attenuated: OR 0.90 (95% CI 0.32–2.56, p = 0.850) for Chemo+IO and 1.86 (95% CI 0.59–5.87, p = 0.292) for Chemo+IO+Targeted vs Chemo, indicating a limited independent effect of regimen after full clinical and biological adjustment.

Lauren classification remained a strong adverse factor. Relative to intestinal-type tumors, diffuse-type and mixed-type tumors had markedly lower odds of MPR, with ORs of 0.04 (95% CI 0.01–0.11) and 0.06 (95% CI 0.01–0.23), respectively (both p < 0.001).

In contrast, PD-L1 emerged as a significant favorable predictor: CPS ≥5 was associated with higher likelihood of MPR compared with CPS <5 (OR 3.92, 95% CI 1.73–8.90, p = 0.001). HER2 positivity and MSI-H status were not significantly related to pathological response in this cohort (HER2 OR 0.95, p = 0.932; MSI-H OR 0.76, p = 0.830) (**Table 5**).

**Table 5:** Extended multivariable logistic regression model including biomarker variables for pathologic response.

Variable	OR	95% CI	p
Regimen: chemo+IO vs chemo	0.90	0.32 – 2.56	0.850
Regimen: chemo+IO+targeted vs chemo	1.86	0.59 – 5.87	0.292
Sex: Male vs Female	0.85	0.27 – 2.69	0.788
Location: Antrum vs Body	0.84	0.31 – 2.27	0.737
Location: Cardia vs Body	0.69	0.25 – 1.89	0.470
Lauren: Diffuse vs Intestinal	0.04	0.01 – 0.11	<0.001
Lauren: Mixed vs Intestinal	0.06	0.01 – 0.23	<0.001
Age	1.00	0.95 – 1.04	0.874
ECOG status	0.52	0.20 – 1.37	0.187
cT	0.99	0.48 – 2.04	0.982
cN	1.06	0.66 – 1.71	0.800
cM: M1 vs M0	2.00	0.50 – 7.95	0.326
HER2: Positive vs Negative	0.95	0.29 – 3.10	0.932
MSI: MSI-H vs MSS	0.76	0.06 – 8.98	0.830
PD-L1: CPS≥5 vs <5	3.92	1.73 – 8.90	0.001

Outcome variable was pathologic response defined as MPR (MPR vs. no-MPR). ORs are adjusted for all variables listed in the table, with the Chemo group as the reference regimen.

### 3.7 Pathological response rates stratified by PD-L1 expression

When stratified by PD-L1 expression, pathological response rates differed across regimens. In the CPS <5 subgroup, MPR rates were 18.2% in the Chemo group, 24.1% in the Chemo+IO group, and 46.2% in the Chemo+IO+Targeted group. In the CPS ≥5 subgroup, overall response rates were higher across three groups (50.0%, 52.2%, and 72.7%, respectively) (**Table 6**).

**Table 6:** Pathologic response by regimen stratified by PD-L1 CPS.

PD-L1 low expression: CPS < 5 (n=113)			PD-L1 high expression: CPS ≥ 5(n=82)		
Regimen	MPR/Total	Proportion	Regimen	MPR/Total	Proportion
chemo	6/33	18.2%	chemo	7/14	50.0%
chemo+IO	13/54	24.1%	chemo+IO	24/46	52.2%
chemo+IO+targeted	12/26	46.2%	chemo+IO+targeted	16/22	72.7%

Data are presented as n (%).

Subgroup logistic regression using the Chemo as reference showed that, in patients with CPS <5, the OR for MPR was 1.43 (95% CI 0.48–4.21, p = 0.520) for Chemo+IO and 3.86 (95% CI 1.19–12.47, p = 0.024) for Chemo+IO+Targeted, indicating a significant benefit of the triple regimen in patients with low PD-L1 expression. In the CPS ≥5 subgroup, the ORs for Chemo+IO and Chemo+IO+Targeted vs Chemo were 1.09 (95% CI 0.33–3.61) and 2.67 (95% CI 0.65–10.88), respectively, although numerically higher, these did not reach statistical significance, likely due to limited sample size (**Supplementary Table S3**).

Overall, high PD-L1 expression is associated with a greater likelihood of pathological response, and in patients with low PD-L1 expression, the triple regimen was associated with a higher MPR rate compared with the other regimens.

### 3.8 Follow-up and survival outcomes (exploratory analysis)

At the current follow-up, exploratory analyses of DFS and OS showed no clear separation of Kaplan–Meier curves among the three treatment regimens up to approximately 36 months (**Supplementary Figures S2**, **S3**). Immunotherapy-containing regimens showed only modest, non-significant trends toward improved DFS and OS compared with Chemo group.

In multivariable Cox models using the Chemo group as reference, the hazard ratios (HR) for DFS and OS in the Chemo+IO group were 0.66 (95% CI 0.33–1.34, p = 0.252) and 0.58 (95% CI 0.23–1.49, p = 0.259), respectively. In the Chemo+IO+Targeted group, the HRs were 1.26 (95% CI 0.59–2.68, p = 0.548) for DFS and 0.94 (95% CI 0.32–2.74, p = 0.909) for OS. None of these associations reached statistical significance, indicating no clear independent survival benefit of any specific regimen at this stage of follow-up.

Among other covariates, cN stage was a consistent adverse prognostic factor for both DFS and OS (HR per increase in cN category 1.53, 95% CI 1.05–2.22, p = 0.026; and 1.68, 95% CI 1.01–2.80, p = 0.048, respectively). Age was positively associated with worse OS (HR per year 1.06, 95% CI 1.01–1.11, p = 0.024), but not with DFS. Gastric antrum location showed a borderline protective effect on DFS compared with gastric body (HR 0.46, 95% CI 0.21–1.00, p = 0.050). ECOG status, cT, cM, and Lauren classification were not significantly associated with DFS or OS (**Supplementary Table S4**).

## 4 Discussion

This study systematically compared the differences in pathological response, safety, and short-term survival among three preoperative systemic strategies (neoadjuvant for cM0; conversion for selected cM1) within a surgically standardized cohort undergoing D2 gastrectomy. Key biomarkers such as Lauren classification, PD-L1, HER2, MSI were included for multivariate and subgroup analysis.

Overall, four main observations emerged. First, pathological response rates increased in a stepwise manner from chemo to Chemo+IO and then to Chemo+IO+Targeted; however, histological subtype and PD-L1 status had a greater impact on pCR/MPR than regimen category itself. Second, Lauren intestinal type and PD-L1 CPS ≥5 were identified as favorable predictors of pCR/MPR, while in PD-L1–low tumors (CPS <5) the triple regimen was associated with higher MPR, suggesting that treatment intensification may mitigate the disadvantage of low PD-L1 status. Third, within the current follow-up period, DFS and OS curves did not diverge significantly, and prognosis remained largely driven by baseline nodal burden and age rather than regimen choice. Fourth, postoperative complication rates and serious AEs were comparable across groups, indicating that regimen intensification did not substantially increase surgical risk. These findings provide complementary real-world evidence supporting triple strategies and biomarker-guided selection of preoperative treatment regimens in gastric cancer undergoing D2 gastrectomy.

In this study, the overall pCR rate was 20% and the combined MPR rate was 40%, with MPR rates of 27.7%, 37.0%, and 58.3% in the Chemo, Chemo+IO, and Chemo+IO+Targeted groups, respectively. In univariate analysis, the triple regimen was associated with a markedly higher likelihood of achieving MPR (OR ≈ 3.7 vs Chemo), and Chemo+IO also showed a numerical advantage. This stepwise pattern is broadly in line with preoperative immunotherapy studies, where adding a PD-1/PD-L1 inhibitor to standard chemotherapy consistently enhances pathological response. KEYNOTE-585 and DANTE demonstrated clear gains in pCR/TRG 1a and downstaging with chemo-immunotherapy, while MATTERHORN further confirmed improvements in both event-free and overall survival with durvalumab plus FLOT (8, 13–15).

Compared with these RCTs, the pathological response rates observed in our three regimens fall within the middle-to-upper range of reported values, but several nuances should be noted. First, our primary pathological endpoint was MPR, whereas key trials typically focus on pCR or TRG 1a; inclusion of MPR inevitably raises the overall response rate. Second, as a real-world cohort, our regimens and targeted components were heterogeneous—some triple regimens incorporated anti-HER2 or anti-angiogenic agents—reflecting higher “treatment intensity” but also potential selection of patients with greater tumor burden or anticipated benefit.

Importantly, once adjusted for Lauren classification, PD-L1, and cTNM stage in multivariable models, the apparent advantage of the triple regimen was substantially attenuated and lost statistical significance, whereas the effects of Lauren intestinal type and PD-L1 (CPS ≥5) became dominant. This pattern suggests that, in routine practice, regimen selection is strongly influenced by underlying tumor biology rather than being quasi-random, and that crude between-group comparisons may overestimate the independent effect of treatment intensity. After adjustment, pathological response appears to be driven primarily by intrinsic tumor sensitivity, which is consistent with the emerging paradigm that emphasizes “treatment regimen × molecular subtype” rather than simple head-to-head comparisons of regimens alone.

This study consistently showed that Lauren intestinal-type tumors were much more likely to achieve pCR/MPR after preoperative treatment, whereas diffuse and mixed types had markedly lower response rates. In both the multivariable and extended models, diffuse and mixed types had ORs of only 0.04–0.06 for pCR/MPR compared with intestinal type. These findings are in line with previous reports that intestinal-type tumors achieve higher pCR rates and better long-term outcomes with preoperative chemotherapy or chemoradiotherapy, whereas diffuse-type—particularly signet-ring cell—tumors are relatively chemoresistant and often display a “cold” immune phenotype (16–19). Recent data also suggest that intestinal-type tumors may be more likely to benefit from preoperative immune-based combinations; our results further support this concept (16, 20).

With respect to immune biomarkers, the extended model confirmed PD-L1 (CPS ≥5) as an independent favorable predictor of pCR/MPR, with an OR close to 4, remaining significant after adjustment for regimen, Lauren classification, HER2, MSI, and other covariates. This is highly consistent with pivotal preoperative immunotherapy trials and meta-analyses showing that patients with high PD-L1 expression derive greater benefit in terms of pathological response and event-free survival (13, 14, 21, 22). PD-L1 has therefore emerged as one of the most mature and actionable biomarkers in the preoperative setting.

A more forward-looking aspect of our work is the exploratory analysis of regimen effects after PD-L1 stratification. In the CPS ≥5 subgroup, MPR rates were high across all three regimens, with only modest numerical advantages for chemo-immunotherapy and the triple regimen over chemotherapy alone. By contrast, in the CPS <5 subgroup, the triple regimen significantly increased MPR, with an OR close to 4, suggesting that treatment intensification may provide additional pathological benefit in tumors with an inherently unfavorable immune phenotype. This observation echoes advanced-disease studies in which anti-HER2 or anti-VEGF agents combined with PD-1 inhibitors retain activity even in PD-L1–low populations (10, 23–25). Because large preoperative RCTs have not yet systematically evaluated triple strategies in PD-L1–low subgroups, our findings should be viewed as hypothesis-generating and warrant prospective validation. moreover, the PD-L1 interaction analysis was underpowered due to limited subgroup sample size.

From a clinical perspective, integrating Lauren classification with PD-L1 status may help construct a more refined risk–benefit framework. For patients with Lauren intestinal-type, PD-L1–high tumors, standard chemotherapy plus immunotherapy may already achieve high pathological response rates, and routine escalation to triple therapy may need careful justification. Conversely, for patients with diffuse or mixed histology and low PD-L1 expression, future trials should prioritize exploring triple or next-generation “immune + targeted” intensified strategies in an attempt to counterbalance their intrinsically unfavorable biology.

Although our study showed a clear advantage in pathological response—particularly with the triple regimen—the DFS and OS curves of the three groups had not separated meaningfully at the current follow-up. Consistent with this, multivariable Cox analyses did not identify the preoperative treatment regimen itself as an independent predictor of DFS/OS, whereas cN stage and age remained the most robust adverse prognostic factors. This pattern echoes the trajectory seen in prior preoperative immunotherapy studies, in which pathological benefit typically precedes demonstrable survival benefit (13, 14, 21, 22).

Clinically, pathological response (MPR, including pCR) reflects the residual viable tumor burden after preoperative therapy and therefore serves as a clinically meaningful early efficacy readout. In gastric cancer, deeper histopathological regression has been associated with a lower recurrence risk and improved long-term outcomes. Biologically, achieving pCR/MPR likely indicates a lower level of minimal residual disease (MRD) (26). In resectable gastric/GEJ adenocarcinoma, postoperative ctDNA positivity (molecular residual disease) has been shown to strongly predict recurrence and survival, providing a mechanistic link between residual tumor burden and long-term outcomes (27). Accordingly, the higher pCR/MPR rates observed with intensified regimens in our cohort may suggest reduced MRD and potential downstream survival benefit, although longer follow-up is required to confirm this.

In KEYNOTE-585, the addition of pembrolizumab substantially increased pCR rates and yielded a modest improvement in EFS, yet the OS HR (~0.8–0.9) did not cross the prespecified significance threshold, and survival advantages were more suggestive than definitive, particularly in PD-L1–high or MSI-H subgroups (13, 21). By contrast, MATTERHORN can be viewed as a “next-stage” trial, in which durvalumab plus FLOT not only improved pathological response and EFS but also achieved a statistically significant OS benefit, with an approximate 22% reduction in the risk of death and generally consistent effects across PD-L1 and nodal subgroups (14). Together, these data underscore that gains in pathological response do not translate into OS in a simple or immediate manner, robust survival benefits usually require larger sample sizes and longer follow-up.

In this context, our negative DFS/OS findings should be interpreted primarily in light of methodological constraints rather than as evidence against the long-term value of immunotherapy or triple regimens. The cohort size was modest, event numbers were limited, and follow-up was relatively short compared with large phase III trials, inevitably restricting statistical power. Our results are therefore best regarded as real-world complementary evidence focused on pathological endpoints and early prognostic signals. Definitive conclusions about long-term survival will require extended follow-up and validation in larger, preferably multi-center, datasets.

In this study, the overall incidence of postoperative complications ranged from 36% to 40% across the three groups, which is highly consistent with the 30–45% reported in D2 gastrectomy and FLOT preoperative treatment series (15, 28, 29). Most events were mild to moderate, such as abdominal distension, pulmonary infection, and intra-abdominal effusion, whereas severe complications (bleeding, anastomotic leakage, severe infection) were infrequent and did not differ significantly between regimens. Similarly, the rates of grade ≥3 AEs, SAEs, and AE-related dose reduction or discontinuation were comparable across groups. As expected, irAEs occurred mainly in the immunotherapy-containing regimens but were generally manageable.

These findings align with the safety profiles reported in DANTE and MATTERHORN, where adding atezolizumab or durvalumab to FLOT did not increase surgery-related morbidity or mortality, and severe postoperative complications remained similar to FLOT alone (8, 14, 30). Taken together, our data support that, under appropriate treatment breaks, nutritional optimization, and standardized postoperative care, preoperative immunotherapy and immunotherapy plus targeted therapy do not substantially increase the surgical risk of D2 gastrectomy.

It should also be noted that this study systematically captured milder complications (e.g., transient abdominal distension and small-volume effusions), which likely elevates the absolute rate of “any complication” and reflects routine clinical practice rather than excess harm. From a decision-making standpoint, severe complications, re-intervention rates, and postoperative mortality are more critical, and these endpoints did not differ between regimens, supporting an acceptable risk–benefit profile for intensified preoperative treatment strategies under standardized management.

This study has several strengths. First, its three-arm real-world design—including Chemo, Chemo+IO, and Chemo+IO+Targeted therapy—captures the contemporary spectrum of preoperative treatment intensity and permits exploratory evaluation of when regimen escalation may be justified. Second, surgical and pathological management were highly standardized, with uniform D2 gastrectomy, an almost universal R0 resection rate, and systematic ypTNM and Becker TRG assessment, thereby reducing confounding from variability in surgical quality. Third, the incorporation of Lauren classification and key immune/molecular biomarkers into multivariable and stratified analyses allowed a more biologically informed interpretation of treatment effects and generated a testable hypothesis that triple regimens may partially mitigate the disadvantage of PD-L1–low tumors.

This study also has several important limitations. First, its single-center, retrospective design inevitably introduces selection and information bias, particularly regarding use of the triple regimen. Treatment choices were influenced by disease burden, patient preference, and economic factors rather than random allocation, resulting in a strong coupling between regimen and baseline characteristics; although multivariable adjustment was performed, residual confounding cannot be excluded. Second, the overall sample size was modest and imbalanced across the three groups, and several biomarker-defined subgroups (e.g. PD-L1, HER2, MSI, Lauren classification) were small, yielding imprecise effect estimates and wide confidence intervals, with some clinically meaningful trends failing to reach statistical significance. Accordingly, PD-L1 subgroup and interaction analyses were exploratory and underpowered, and should be considered hypothesis-generating. Third, targeted agents in the triple-regimen group were heterogeneous (e.g., anti-HER2 and anti-angiogenic therapies) and were analyzed together; thus, no agent-specific efficacy or safety conclusions can be drawn. This reflects real-world practice but limits agent-specific inferences. Fourth, follow-up duration and event numbers were limited, resulting in restricted statistical power for DFS/OS and an evidence level that remains lower than that of mature phase III trials such as MATTERHORN.

These limitations point to several directions for future research. The “biomarker + treatment intensity” hypothesis generated in this study should be tested in prospective multicenter cohorts, particularly in patients with Lauren diffuse/mixed histology, low PD-L1 expression, or HER2-negative tumors with high tumor burden, to more rigorously evaluate the benefit of triple and next-generation immune-based combinations on both pathological and survival outcomes. Integration of multimodal data—including imaging radiomics, digital pathology, circulating tumor DNA, and multi-omics—will be crucial for building robust models to predict pCR/MPR and minimal residual disease risk, thereby guiding individualized preoperative treatment strategies (31, 32). In parallel, extended follow-up and collaborative analyses with external cohorts are needed to clarify the relationship between pathological response and long-term outcomes.

## 5 Conclusion

In this real-world cohort of D2 gastrectomy, preoperative treatment regimens incorporating immunotherapy—especially chemotherapy combined with immunotherapy and targeted therapy—were associated with higher pathological response rates without a meaningful increase in risk. After adjustment, however, regimen choice had only a modest independent effect, while Lauren subtype and PD-L1 expression were the main determinants of pCR/MPR: intestinal-type, PD-L1–high tumors responded best, and the triple regimen was associated with higher response in PD-L1–low disease. No significant differences in DFS or OS were observed at the current follow-up, underscoring the need for prospective, biomarker-guided multicenter studies to validate these findings and optimize preoperative treatment strategies.

## Data Availability

The original contributions presented in the study are included in the article/**Supplementary Material**. Further inquiries can be directed to the corresponding author.
