# Supplementary material for: Pathologic response and safety of preoperative treatment regimens in gastric cancer undergoing D2 gastrectomy: a real-world cohort study
Source: Front Immunol. 2026 Jan 29;17:1766793. doi: 10.3389/fimmu.2026.1766793 (PMC12893969; doi:10.3389/fimmu.2026.1766793)
Supplement: Supplementary file 1 [file DataSheet1.docx]

**Supplementary Table 1. Preoperative treatment exposure and adverse events by regimen**

| Variable | | Total  (n=195) | Chemo  (n=47) | Chemo+IO  (n=100) | Chemo+IO+Targeted (n=48) | *p* |
| --- | --- | --- | --- | --- | --- | --- |
| Treatment Process | preoperative cycles,  median ( IQR) | 4 (3,4) | 4 (4,4) | 4 (3,4) | 4 (3,4) | 0.175 |
|  | Interval from treatment to surgery, weeks, median ( IQR) | 14.0 (11.5,16.0) | 16.0 (12.0,17.5) | 14.0 (11.0,16.0) | 14.0 (11.8,15.2) | 0.054 |
| Adverse Events | Any AE (≥Grade 1), n (%) | 188 (96.4%) | 46 (97.9%) | 95 (95.0%) | 47 (97.9%) | 0.554 |
|  | Grade ≥3 AE, n (%) | 73 (37.4%) | 14 (29.8%) | 38 (38.0%) | 21 (43.8%) | 0.367 |
|  | Immune-related AE (irAE), n (%) | 32 (16.4%) | 0 (0.0%) | 20 (20.0%) | 12 (25.0%) | 0.002 |
|  | Serious AE (SAE), n (%) | 24 (12.3%) | 5 (10.6%) | 12 (12.0%) | 7 (14.6%) | 0.835 |
|  | Treatment dose reduction or interruption due to AE, n (%) | 59 (30.3%) | 16 (34.0%) | 28 (28.0%) | 15 (31.2%) | 0.747 |
|  | Permanent discontinuation of treatment due to AE, n (%) | 11 (5.6%) | 1 (2.1%) | 6 (6.0%) | 4 (8.3%) | 0.413 |

Data are presented as median (IQR) or n (%). p values refer to overall comparisons among the three groups (Kruskal–Wallis test for continuous variables and χ² test for categorical variables).

**Supplementary Table 2. Univariable logistic regression for the association between treatment regimen and pathologic response**

| Regimen | OR | 95% CI | p |
| --- | --- | --- | --- |
| chemo+IO vs chemo | 1.54 | 0.72–3.28 | 0.267 |
| chemo+IO+targeted vs chemo | 3.66 | 1.55–8.64 | 0.003 |

Outcome variable was pathologic response defined as MPR (MPR vs. no-MPR). ORs are calculated with the Chemo group as the reference.

**Supplementary Table 3. Subgroup logistic regression by PD-L1 CPS for the association between regimen and pathologic response**

| Variable | | OR | 95% CI | p |
| --- | --- | --- | --- | --- |
| PD-L1 CPS<5 | chemo+IO vs chemo | 1.43 | 0.48–4.21 | 0.520 |
|  | chemo+IO+targeted vs chemo | 3.86 | 1.19–12.47 | 0.024 |
| PD-L1 CPS≥5 | chemo+IO vs chemo | 1.09 | 0.33–3.61 | 0.887 |
|  | chemo+IO+targeted vs chemo | 2.67 | 0.65–10.88 | 0.172 |

Outcome variable was pathologic response defined as MPR (MPR vs. no-MPR). ORs and 95% CIs were estimated separately within each PD-L1 CPS subgroup, with the Chemo group as the reference.

**Supplementary Table 4. Multivariable Cox regression analysis for disease-free survival (DFS) and overall survival (OS).**

| Variable | DFS | | | OS | | |
| --- | --- | --- | --- | --- | --- | --- |
|  | HR | 95% CI | p | HR | 95% CI | p |
| Regimen: chemo+IO vs chemo | 0.66 | 0.33–1.34 | 0.252 | 0.58 | 0.23–1.49 | 0.259 |
| Regimen: chemo+IO+targeted vs chemo | 1.26 | 0.59–2.68 | 0.548 | 0.94 | 0.32–2.74 | 0.909 |
| Sex: Female vs Male | 0.84 | 0.32–2.18 | 0.717 | 0.27 | 0.04–2.08 | 0.208 |
| Age (per year) | 1.01 | 0.98–1.05 | 0.458 | 1.06 | 1.01–1.11 | 0.024 |
| ECOG (per 1 point) | 1.57 | 0.80–3.08 | 0.187 | 1.44 | 0.60–3.45 | 0.411 |
| cT (per stage) | 1.06 | 0.59–1.91 | 0.845 | 1.07 | 0.47–2.40 | 0.876 |
| cN (per stage) | 1.53 | 1.05–2.22 | 0.026 | 1.68 | 1.01–2.80 | 0.048 |
| cM: M1 vs M0 | 0.45 | 0.13–1.49 | 0.191 | 0.36 | 0.05–2.76 | 0.328 |
| Location: Antrum vs Body | 0.46 | 0.21–1.00 | 0.050 | 0.60 | 0.19–1.90 | 0.387 |
| Location: Cardia vs Body | 0.76 | 0.39–1.51 | 0.439 | 1.24 | 0.48–3.20 | 0.660 |
| Lauren: mixed vs diffuse | 1.03 | 0.44–2.44 | 0.943 | 0.99 | 0.29–3.36 | 0.987 |
| Lauren: intestinal vs diffuse | 0.70 | 0.35–1.40 | 0.313 | 1.02 | 0.39–2.67 | 0.973 |

**Supplementary Table S1. Sensitivity Analysis (After Excluding cM1)**

| Variable | Total  (n=178) | Chemo  (n=42) | Chemo+IO  (n=92) | Chemo+IO+Targeted (n=44) | *p* |
| --- | --- | --- | --- | --- | --- |
| Pathological Response, n (%) |  |  |  |  | 0.012 |
| MPR，n (%) | 70 (39.3%) | 11 (26.2%) | 34 (37.0%) | 25 (56.8%) |  |

| Variable | OR | 95% CI | p |
| --- | --- | --- | --- |
| Regimen: chemo+IO vs chemo | 1.10 | (0.39–3.14) | 0.858 |
| Regimen: chemo+IO+targeted vs chemo | 1.73 | (0.53–5.63) | 0.366 |
| Sex: Male vs Female | 1.05 | (0.35–3.21) | 0.928 |
| Location: Antrum vs Body | 1.07 | (0.39–2.90) | 0.899 |
| Location: Cardia vs Body | 0.59 | (0.22–1.61) | 0.302 |
| Lauren: Diffuse vs Intestinal | 0.05 | (0.02–0.14) | <0.001 |
| Lauren: Mixed vs Intestinal | 0.04 | (0.01–0.20) | <0.001 |
| Age | 0.95 | (0.29–3.09) | 0.930 |
| ECOG status | 0.41 | (0.16–1.04) | 0.060 |
| cT | 0.77 | (0.37–1.59) | 0.484 |
| cN | 1.07 | (0.67–1.70) | 0.776 |

**Supplementary Table S2. Composition of chemotherapy, immunotherapy and targeted agents by regimen group**

**S2A. Immune checkpoint inhibitors (ICIs)**

| Regimen group | ICI agent | Class/target | n / total (%) |
| --- | --- | --- | --- |
| Chemo+IO | Cadonilimab | PD-1/CTLA-4 bispecific antibody | 55 / 100 (55.0%) |
| Chemo+IO | Sindilimab | PD-1 inhibitor | 39 / 100 (39.0%) |
| Chemo+IO | Tislelizumab | PD-1 inhibitor | 3 / 100 (3.0%) |
| Chemo+IO | Serplulimab | PD-1 inhibitor | 2 / 100 (2.0%) |
| Chemo+IO | Camrelizumab | PD-1 inhibitor | 1 / 100 (1.0%) |
| Chemo+IO+Targeted | Sindilimab | PD-1 inhibitor | 40 / 48 (83.3%) |
| Chemo+IO+Targeted | Serplulimab | PD-1 inhibitor | 4 / 48 (8.3%) |
| Chemo+IO+Targeted | Cadonilimab | PD-1/CTLA-4 bispecific antibody | 2 / 48 (4.2%) |
| Chemo+IO+Targeted | Camrelizumab | PD-1 inhibitor | 2 / 48 (4.2%) |

**S2B. Targeted agents (Chemo+IO+Targeted group only, n=48)**

| Regimen group | Targeted agent | Class/target | n / total (%) | Mechanism grouping |
| --- | --- | --- | --- | --- |
| Chemo+IO+Targeted | Apatinib | VEGFR2 TKI | 35 / 48 (72.9%) | Anti-angiogenic |
| Chemo+IO+Targeted | Ramucirumab | Anti-VEGFR2 mAb | 4 / 48 (8.3%) | Anti-angiogenic |
| Chemo+IO+Targeted | Trastuzumab | Anti-HER2 mAb | 7 / 48 (14.6%) | Anti-HER2 |
| Chemo+IO+Targeted | Disitamab vedotin | HER2 ADC | 2 / 48 (4.2%) | Anti-HER2 |

**S2C. Chemotherapy backbones by regimen group**

| Regimen group | FLOT, n / total (%) | SOX, n / total (%) |
| --- | --- | --- |
| Chemo (n=47) | 39 / 47 (83.0%) | 8 / 47 (17.0%) |
| Chemo+IO (n=100) | 50 / 100 (50.0%) | 50 / 100 (50.0%) |
| Chemo+IO+Targeted (n=48) | 25 / 48 (52.1%) | 23 / 48 (47.9%) |

Chemotherapy served as the backbone for all groups and consisted primarily of platinum plus fluoropyrimidine regimens, mainly SOX and FLOT. Abbreviations: ICI, immune checkpoint inhibitor; TKI, tyrosine kinase inhibitor; mAb, monoclonal antibody; ADC, antibody–drug conjugate; VEGFR2, vascular endothelial growth factor receptor 2.
