# Supplementary material for: Pathologic response and safety of preoperative treatment regimens in gastric cancer undergoing D2 gastrectomy: a real-world cohort study
Source: Front Immunol. 2026 Jan 29;17:1766793. doi: 10.3389/fimmu.2026.1766793 (PMC12893969; doi:10.3389/fimmu.2026.1766793)
Supplement: Supplementary file 2 [file DataSheet2.docx]

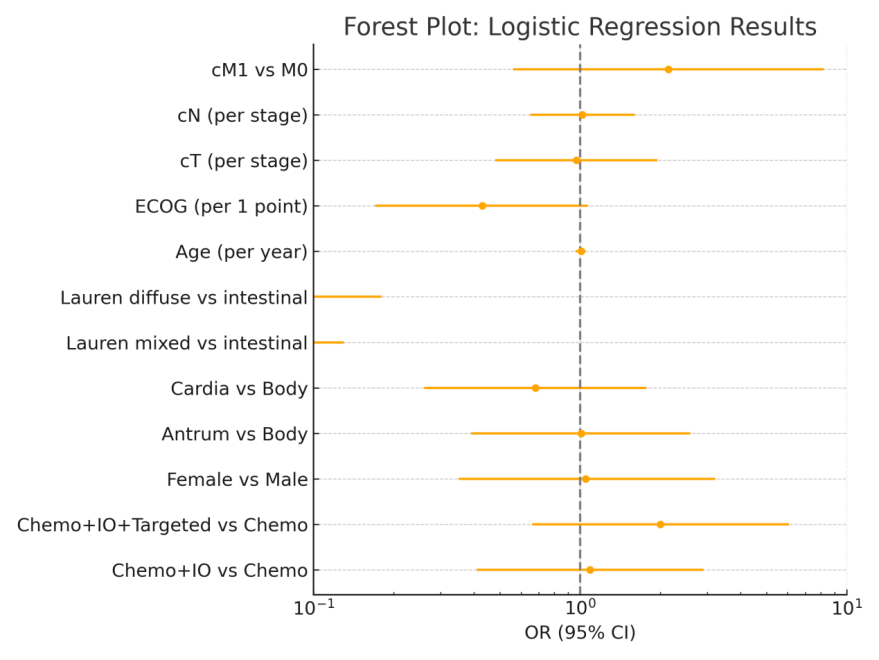


**Supplementary Figure 1. Forest plot of multivariable logistic regression for pathologic response**

**
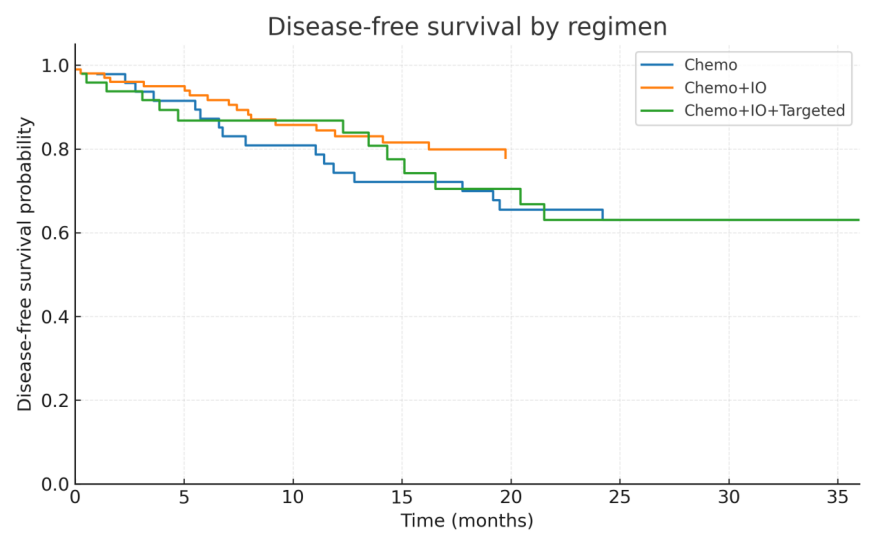
**

**Supplementary Figure 2. Kaplan–Meier curves for disease-free survival by neoadjuvant regimen**

**
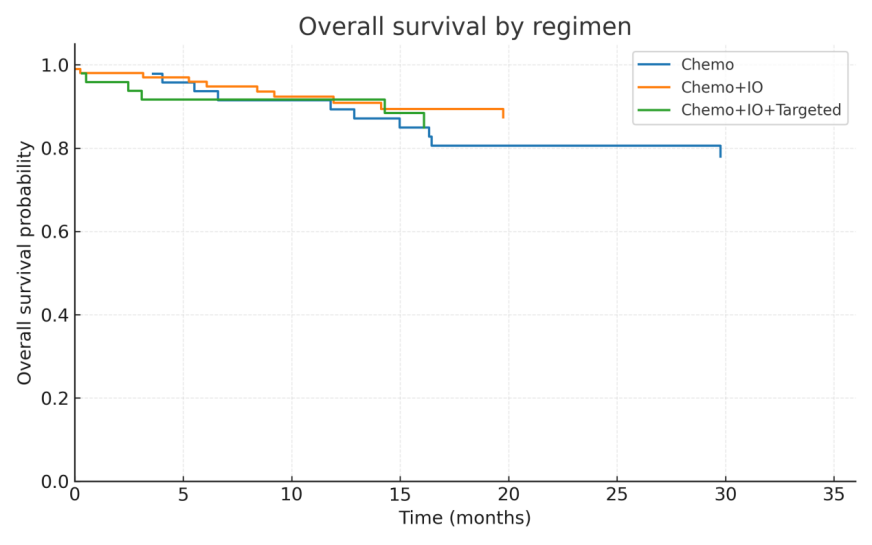
**

**Supplementary Figure 3. Kaplan–Meier curves for overall survival by neoadjuvant regimen**
